# The effect of micro-learning on trauma care knowledge and learning satisfaction in nursing students

**DOI:** 10.1186/s12909-023-04609-2

**Published:** 2023-09-01

**Authors:** Hossein Haghighat, Maryam Shiri, Mohammad Esmaeili Abdar, Seyedeh Soghra Taher Harikandee, Zahra Tayebi

**Affiliations:** 1 School of Nursing, Alborz University of Medical Sciences, Karaj, IR Iran; 2 Medical education department, school of medicine, Tehran University of medical sciences, Tehran, Iran; 3 School of Nursing, Kashan University of Medical Sciences, Kashan, IR Iran; 4 Social Determinants of Health Research Center, Alborz University of Medical Sciences, Karaj, IR Iran

**Keywords:** Micro learning, Trauma, Nursing students, Emergency, ATLS

## Abstract

**Background:**

Despite the fact that there are few formal trauma training courses for nurses, they play an important role in the care of trauma patients. This study aims to investigate the effect of micro-learning on the knowledge of managing trauma patients and learning satisfaction in nursing students.

**Methods:**

The convenience sampling method was used to enroll 30 final-year nursing students from Alborz University of Medical Sciences in this quasi experimental One-group pretest -posttest design. The educational content was created and repeated 4 times over the course of 36 days using a micro-learning approach through whiteboard animations, video casts, and live videos. MCQ scenario-based exam was used to assess participants’ knowledge of trauma in three phases: pretest, immediately following the intervention, and one month after the end of the educational program. An e-learning satisfaction psychometric questionnaire was used to measure satisfaction.

**Results:**

The mean knowledge score 1 month after the intervention did not differ significantly from the score immediately after the intervention (p = 1), but there was a significant relationship between the mean knowledge score immediately after the intervention and before that (p = 0.047). Demographic variables and knowledge of trauma management did not differ statistically significant. The majority of students were pleased with how the course was implemented (5.64).

**Conclusion:**

The use of micro-learning has a positive effect on the promotion and retention of knowledge of trauma care, as well as increasing nursing students’ satisfaction. Micro-learning is proposed as a new educational approach that can be used as a complementary or as a stand-alone method to convey important educational concepts and increase learner satisfaction.

## Key point

1. Micro-learning approach has a positive effect on promotion of the trauma care knowledge.
2. Repetition of small pieces of educational contents through microlearning is effective in retaining knowledge.
3. Students were satisfied with the structure and format of the micro-learning course.

## Introduction

Trauma is currently the most common cause of death in children and young adults worldwide and one of the major contributors to disability [1]. The World Health Organization estimated that 5 to 8.5 million deaths worldwide occur each year as a result of trauma. By 2030, there will be a 40% increase in these deaths, making trauma a top priority. Iran also had one of the highest rates of fatal traffic accidents, with 1.34 per 100,000 persons in 2013 and 1.32 in 2014 as a result of trauma and traffic accidents [2].

Nurses, as one of the treatment team’s main pillars, play an important role in effective trauma management. Optimal nursing care can reduce problems caused by long-term disabilities as well as trauma-related mortality. However, the findings of studies on nurses’ ability to care for trauma patients show a lack of knowledge and skills among nurses in this field [3]. Nursing managers came to the conclusion, based on the Advance Trauma Life Support course, that educational programs should be created and implemented to strengthen nurses’ weak points and enhance the standard of nursing care and trauma patient safety [4]. The American College of Surgeons recommends using the Advanced Trauma Life Support training to treat trauma patients [5]. Research has shown that ATLS trauma standards improve treatment options, lessen problems associated with long-term disability, and reduce trauma-related mortality [6, 7].

Traditional methods are still used as a teaching format for health-therapeutic topics despite considerable improvements in the field of teaching approach, as the way that educational information is presented is vital to its success [8–10]. In recent years, however, the arrival of new technologies has given rise to opportunities to offer creative ways to teaching. Continuous and self-directed learning, as well as modern educational methods, are commonly used to improve and reinforce clinical decision-making skills [11, 12].

Micro-learning is one of the cutting-edge teaching-learning methodologies in the era of digital media, and it is crucial to ongoing education. Micro-learning is the step-by-step learning of a small learning nugget [13]. This learning theory gives concentrated, applicable, and brief knowledge that is easy to understand and analyze, and it lays a major emphasis on the creativity, adaptability, and capacity of learners [14]. According to specialists in organizational training, micro-learning is a practical and effective method for enhancing knowledge and skills as well as raising competency inside an organization. This learning approach may be communicated through text messages, podcasts, brief movies, infographics, motion graphics, social media, and other channels [13].

In a systematic review study on the efficacy of mobile micro-learning, Lee found that it not only enhances knowledge and raises learner motivation, but also results in the retention and preservation of knowledge and abilities [15].

A brief review of the undergraduate nursing curriculum demonstrates the significance of trauma education for nursing students. The favorable attitude of current generations of students toward digital media highlights the need of utilizing new technology-based solutions. The goal of this study was to see how the micro-learning method affected final-year nursing students’ understanding of managing trauma patients based on the Advanced Life Support course at…… University of Medical Sciences in the academic year 2022.

## Methods

The current study was One-group pretest -posttest design. Through convenience sampling method, 30 of the final-year nursing students at Alborz University of Medical Sciences were included in the study. GPOWER software was used to calculate the sample size. Considering the first type error of 5%, the power of test 80% and the average effect size of 0.4 (dependent variable: trauma care knowlege), the sample size was estimated to be 30 people. The inclusion criteria were passing 1.5 theory units of emergency nursing and 1 unit of trauma emergency placement, as well as a willingness to participate in the study. The student who did not study more than 10% of the content was excluded from the study due to non-compliance with the educational program during the course. Students who had concurrently received a related training course were also excluded from the study. It was made clear to the students that participation in the study is voluntary, and their written consent was obtained.

The first and second levels of Kirkpatrick’s four-level model were used to evaluate the effectiveness of this course. At the reaction level, participants’ feelings and satisfaction with the program were evaluated using a psychometric questionnaire of e-learning satisfaction. Student Learning and Satisfaction in Online Learning Environments Instrument (SLS-OLE) was used. This questionnaire contains 34 questions in six domains of “course structure, learner interaction, student engagement, instructor presence, student satisfaction, perceived learning” with seven-point Likert from strongly disagree [1] to strongly agree [7]. After obtaining permission from the developer of scale [16] it was translated to Persian by the researcher, and then its face and content validity were evaluated by 10 professors in the fields of nursing and medical education. The CVI and CVR of items calculated and the items were valid (CVI > 0.7, CVR > 0.62). Qualitative content validity was also performed and according to the nature of the intervention of the study, some items were removed or revised and a shortened form of the instrument was used. In this study, the internal consistency of SLS-OLE was assessed by Cronbach’s alpha; 0.78 for the course structure, 0.82 for learner interaction, 0.72 for student engagement, 0.78 for instructor presence, 0.94 for students’ satisfaction and 0.88 for the perceived learning. An online scenario-based MCQ test was created to evaluate the second level (learning) of Kirkpatrick’s model. This form contains 20 multiple-choice questions based on the ATLS course, text books, and the NCLEX RN tests. Ten faculty members in the field of emergency and nursing evaluated the face and content validity of questions. They were asked to give their opinion regarding the grammar, the use of appropriate words, clarity and simplicity as well as the relevance of the questions. The necessary changes were applied based on their comments. The test’s face validity was assessed in a pilot group of 10 emergency nurses. Students who met the inclusion criteria were invited to What’s App group, and the study protocol was explained. The students were evaluated three times: once before the course, once immediately after, and once one month later. To minimize the carry-over effect, a two-week gap was included between the pre-test and the starting the course. The desired trauma management content was extracted from the Advanced Trauma Nursing Course [16] (Table 1). This course is extracted from the main content of the Advanced Trauma Life Support that is provided by the American Association of Surgeons for the purpose of teaching acute trauma management.

**Table 1:** Advanced Trauma Nursing Course titles

Suggested topics for the nursing trauma course	
1. Epidemiology and mechanism of injury and trauma 2. Airway management 3. Shock 4. Chest and abdomen trauma 5. Head trauma 6. Spinal trauma	

Content production was done through Video Scribe, Canva, Inshot and Camtasia software in the form of whiteboard animation, live video, and video casts. The content format was chosen based on the topic characteristics. For each of the topics mentioned in Table 1, one content was produced and designed only to achieve the goals of that topic. The duration of the content varied between 3 and 7 min which presented through WhatsApp messaging network and local learning management system (NAVID).Our content was repeated on days 1, 2, 6, and 31 according to Hermann Ebbinghaus’ theory of the forgetting curve [17]. A protocol was created prior to the start of the study and distributed to the participants with full details via the learning management system (NAVID). The research team also created an audiocast where they went into great detail about the study’s methodology and highlighted any potential difficulties.

Descriptive statistics (mean, and standard deviation) and inferential statistics (Chi-square, Fisher’s exact tests, and univariate and multivariate covariance analysis) were used in the data analysis. SPSS software version 26 was used to process the data.

### Ethical consideration

This project was approved in research committee of Alborz university of Medical sciences with code of IR.ABZUM.REC.1400.313.

## Results

The majority of the students (66.7%) were female and had an average age of 22.90 0.92 years. 6.7% of students had completed the trauma-related course outside the college and 93.3% had a job experiences (Table 2).

**Table 2:** Demographic characteristics of nursing students

Demographic characteristics		Number	%
Gender	female	20	66.7
	male	10	33.3
Academic semester	Semester 7	17	56.7
	Semester 8	13	43.3
The number of trauma emergency units completed	1 academic credit	12	40
Work experience in general clinical departments	2 academic credit	18	60
Taking a trauma-related course outside of college	yes	28	93.3
	no	2	6.7
	yes	2	6.7
	no	28	93.3
Age (year); (standard deviation)	22.90 (0.92)		
Minimum-maximum	21–25		
GPA; (standard deviation)	16.74 (1.09)		
Minimum-maximum	18.70-13.22		

The average score of trauma care knowledge before micro-learning training (10.60 ± 2.19), immediately after (11.76 ± 1.73), and one month after (11.73 ± 1.08) was obtained (Table 3), and the effect size was 0.164(F = 5.691, P = 0.012). Considering that trauma care knowledge was significant in three stages based on repeated measurement, the times were compared based on the Bonferroni pairwise comparison test to determine at which time the difference is significant(Table 4).

**Table 3:** The results of trauma care knowledge before, immediately after and one month after the intervention

Variable	Before intervention mean ± standard deviation	After intervention mean ± standard deviation	One months after intervention mean ± standard deviation		Effect size
Trauma care knowledge	10.6 ± 2.19	11.76 ± 1.73	11.73 ± 1.08	F = 5.691 P = 0.012	0.164

**Table 4:** The results of the pairwise comparison test of the trauma care knowledge scores

Variable		mean difference	p-value
Before intervention	Immediately after the intervention One month after the intervention	-1.167 -1.133	0.047 0.047
Immediately after the intervention		0.033	1

Table 3 shows that there was a significant difference in nursing students’ knowledge of trauma patient management immediately after the intervention (P = 0.047) and one month after (P = 0.047) compared to before the intervention. There is no significant difference in the knowledge score of nursing students’ management of trauma patients one month after the intervention versus immediately after (P = 1), indicating the effectiveness of the micro-learning method on the retention of nursing students’ knowledge of managing trauma patients (Fig. no 1).

**Fig. 1:**
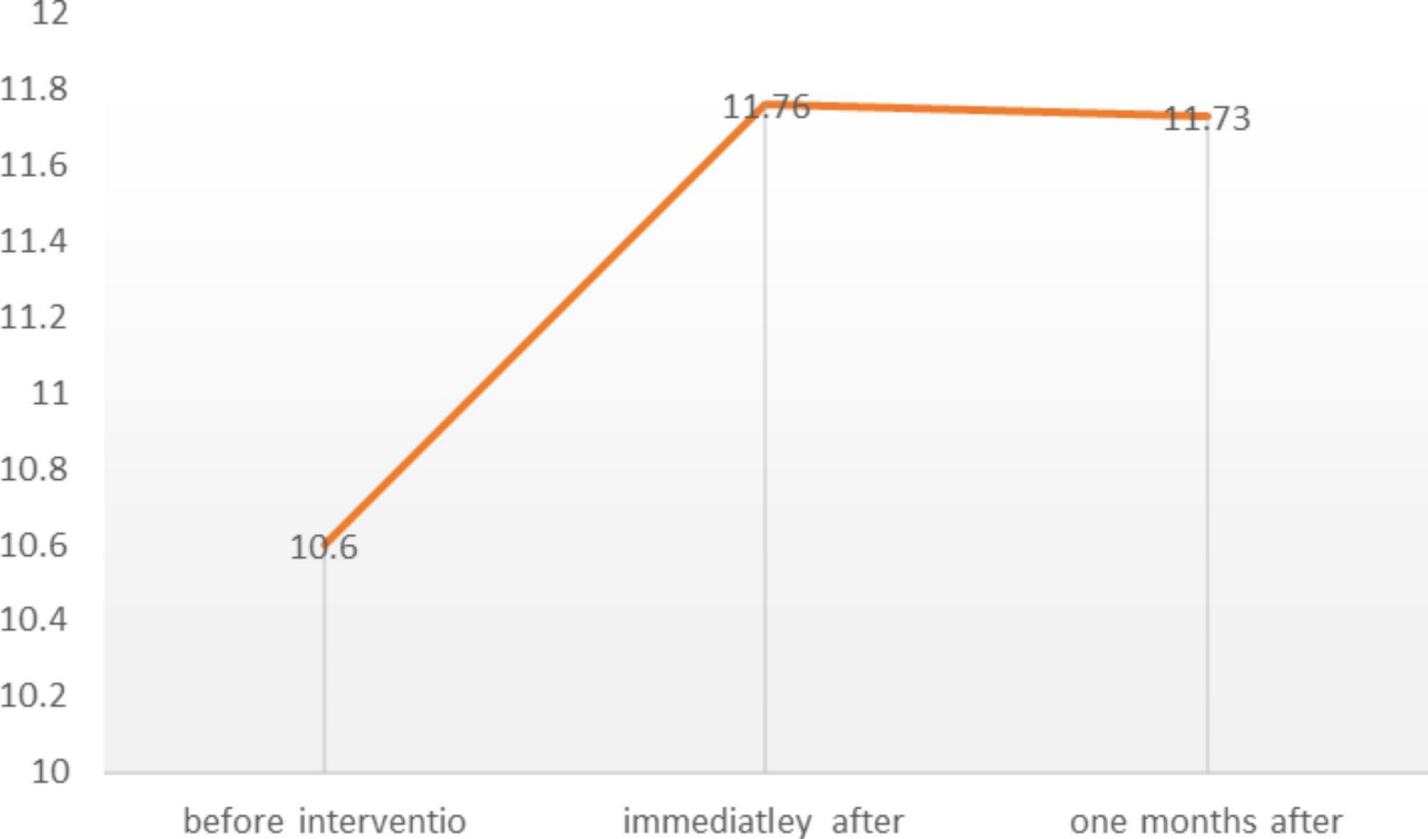
Comparing Knowledge score of trauma patient management in nursing students

Independent t-tests and Pearson’s correlation coefficient were used to investigate the relationship between demographic characteristics and the level of trauma management knowledge. The results showed that there is no statistically significant difference between the demographic variables and the level of trauma management knowledge immediately after (P > 0.05) and one month after the intervention (P > 0.05). Therefore, the micro-learning method has the same effectiveness in increasing the level of trauma management in the levels of demographic variables.

**Table 5:** The effect of time and demographic variables on the level of trauma care knowledge

variable	df	Average of squares	F	P-Value	Effect size
Gender	1	0.482	0.160	0.690	0.002
Academic semester	1	7.049	2.337	0.130	0.028
The number of trauma emergency units completed	1	1.998	0.663	0.418	0.008
Age	1	0.019	0.006	0.937	0.000
GPA	1	3.254	1.079	0.302	0.013
Time	2	13.233	4.388	0.015	0.097

In addition, mixed variance analysis was used to investigate the effect of time and demographic variables on trauma management knowledge (Table 5). The results revealed that time had a significant effect on trauma management knowledge (P = 0.05). As a result, the use of the micro-learning method has been effective in increasing nursing students’ knowledge of trauma management.

**Table 6:** Learning satisfaction of nursing students

Items	Mean	Standard deviation
1) The instruction on how to participate the student in the course was fully provided.	6.57	0.81
2) The implementation of this course did not have a logical procedure.	5.95	1.59
3) The design of the course was not properly organized.	6.23	1.09
4) The purpose of this training course was well explained.	6.14	1.38
5) In this course, I interacted with other students.	4.23	2.14
6) I interacted with the course instructor in this course.	5.61	1.68
7) I used to discuss the educational content of this course outside the class.	4.33	1.82
8) I did not participate effectively and actively in the activities of the training course.	5.33	1.46
9) I am satisfied that I experienced this training course.	6.33	1.11
10) I do not recommend this course to other students.	5.95	1.28
11) I am satisfied with the level of students’ interaction in this training course.	5.33	1.79
12) I am satisfied with what I learned in this training course.	6.14	1.19
13) I am satisfied with the course instructor.	6.14	1.27
14) I am satisfied with the content of the course.	5.52	1.80
15) In this course, I learned less than I expected.	4.28	1.87
16) I learned skills that will help me in the future.	6.09	1.13
17) This training course helped my professional development.	5.76	1.41
**Overall satisfaction**	**5.64**	**0.66**

Table 6. Shows the average satisfaction of the research samples with the structure and implementation of the micro-learning course. The average satisfaction was 5.64 (standard deviation = 0.66). Considering that this value is higher than the average value of 4 (based on the 7-option Likert scale from 1 to 7), the students’ satisfaction with the educational method has an acceptable level. The results show that the average of all 17 satisfaction indicators was higher than the average value of 4. The results show that the highest average satisfaction of nursing students was related to the item “Instructions related to the student’s participation in the course were fully provided” (6.57 ± 0.81) and the lowest average was related to item, “I interacted with other students in this course” (4.23 ± 2.14). The students were satisfied with attending the course and the material they learned and declared that the content they learned is helpful in their professional future.

## Discussion

This study is the first interventional study with the goal of evaluating the impact of micro-learning on Iranian nursing students’ knowledge of trauma care.

Trauma care is often hindered by the difficulty of pinpointing the effects of various factors on a patient’s clinical outcomes. To address this, the development and implementation of trauma-related educational programs has been a priority. Studies have shown that Advanced Trauma Life Support Care programs have been successful in improving patient outcomes [18].

The results of the current study show there is a significant difference between the mean knowledge scores for trauma management before and after the intervention. The effectiveness of microlearning among medical students has been evaluated in numerous situations. Zarshenas and colleagues [19] confirmed to the success of the micro-learning strategy in enhancing nursing students’ knowledge about vaccinations. The final exam scores of the medical students in the Sozmen study [20] who took part in the microlearning-based training programs during the Covid-19 pandemic performed better than the nonparticipating students. Additionally, research respondents were more confident in their ability to carry out their everyday tasks. The findings of Khoshnoudi Far et al. ‘s study also showed that nurses who used the electronic learning approach were more knowledgeable, more skilled, and more satisfied with their training in cardiopulmonary resuscitation than nurses who used the traditional training methods [21].

Despite the fact that electronic learning programs provide a flexible teaching approach, MacDonald and colleagues’ research shown that this learning style, when combined with conventional teaching methods, offers a superior learning style [22].

Microlearning has also been shown to be successful in studies that are not related to medicine. Dabbagh and Heydarpour’s investigation examining the effect of micro-learning on the degree of physics learning involved ninth grade students at a high school. The results showed that, in comparison to the conventional approach, microlearning improves students’ levels of learning. Additionally, traditional learning was more likely than microlearning to lead to children receiving low grades [23].

One of the major issues with developing educational programs is the retention of learning after the course is over. According to Hermann Ebbinghaus’ hypothesis of the forgetting curve, less than 20% of knowledge obtained in the traditional manner will be maintained permanently after a month. According to Ebbinghaus, the proportion of memorization grows with each repetition of the subject and reaches a maximum with five repetitions [17]. The findings of our study provides support to Ebbinghaus’ theory because they show that learning remained stable one month after the intervention because the mean score of nursing students’ knowledge did not significantly differ from the score right after the intervention (p = 1). The grades in Tippet’s study, which examined knowledge retention three months after completing the face-to-face advanced trauma nursing care course, were not significantly different from the scores before enrolling in the course and indicated that knowledge retention was poor; however, the study’s findings revealed a significant impact of taking the training course on the trauma knowledge level of emergency nurses right after the course was finished [18].

According to a review study’s findings, applying mobile devices for microlearning to enhance information retention and job performance speeds up learning overall since it helps students learn more quickly [24].; A month following the intervention may not be the most appropriate period for such an assessment, thus more research is needed to determine how well knowledge is retained.

After using the microlearning method, the current study demonstrated no statistically significant relationship between demographic factors and trauma management knowledge (P > 0.05). Therefore, the micro-learning strategy is equally effective at enhancing knowledge of trauma management and trauma care across demographic characteristics. However, the type of the variables and the characteristics of the research populations were two of the most significant aspects in this study’s lack of effect from demographic variables. For instance, a study by Heydari et al. found that the preparation of emergency nurses in caring for trauma patients has a significant relationship with education, age, and work experience, which contradicts our findings. This is because in their study, a completely different range of nursing education categories, including nursing assistant, bachelor’s, and master’s degrees, were present, whereas in the current study, all nursing education categories were present [4]. Additionally, the age in the current study has a relatively low standard deviation when compared to Heydari’s study. Their participants’ average age was 31.06 ± 5.97, which is significantly older than the average age in our study and may have affected the findings. Additionally, because the students’ clinical work experience is limited, it is unlikely to significantly affect our study; job experience ought to be enough to have an impact on knowledge. The research of RahimKhanali supports these influencing factors as well. Their results revealed that there was a relationship between age and education level with skills [1].

The average of all 17 satisfaction measures is higher than the national average, which is 4, according to the student leaning satisfaction, which is regarded favorable. The results show that “Instructions related to the student’s participation in the course were fully provided” is related to the highest average satisfaction, while “In this training course, I interacted with other students” is related to the lowest average satisfaction.

E-learning courses are generally well-liked, according to studies. For instance, Hegerius and associates reported a very high level of overall satisfaction with the course, with more than 90% of the study respondents rating their satisfaction as “excellent” or “good” in a study that examined micro-learning-based programs in the field of pharmaceutical product care [25]. Emergency nurses in Yazdan Nik et al. study.’s [26] were also pleased with e-learning programs Kosen and Evi (2021) [27] concluded that micro-learning is associated with a significant increase in student participation, satisfaction, and performance in a study aimed at investigating the effectiveness of micro-learning as a tool to increase students’ interaction and learning in universities in Australia and Malaysia.

### Limitation

Production of content took time and required a variety of skills. The length of the knowledge retention evaluation was one month due to the time constraint for the project’s implementation.

## Conclusion

The findings of this quasi-experimental study demonstrated that micro-learning significantly improves nursing students’ knowledge of trauma care. The interesting aspect of this study is how micro-learning affects memorization and retention of newly learned information within a month after the training course’s completion, facilitating the transfer of information from short-term memory to long-term memory. According to the course satisfaction survey findings, the majority of the analyzed items relating the course structure and implementation received high ratings from the participants. The results of this study offer valuable information to educational managers of academic institutions as well as continuing and in-service education facilities. Micro-learning training packages can be offered as standalone courses or as supplements to face-to-face instruction in a variety of medical science fields.

## Data Availability

Complete written data are not provided for publication due to language barriers (the language used is Persian, which is the native language of the participants and the authors). However, the corresponding author is prepared to provide the data for our peer review process if needed.
